# A Case Report of Membranoproliferative Glomerulonephritis: Infection‐Related or Immune‐Related?

**DOI:** 10.1002/ccr3.70088

**Published:** 2025-01-06

**Authors:** Ayman Azhary, Mohammed Taha, Nooh Mohamed Hajhamed, Salahaldeen Ismail Mohammed, Nouh Saad Mohamed, Waleed Azhary Sir Alkhatim

**Affiliations:** ^1^ Department of Medical Microbiology, Faculty of Medical Laboratory Sciences Omdurman Islamic University Khartoum Sudan; ^2^ Molecular Biology Unit Sirius Training and Research Centre Khartoum Sudan; ^3^ Department of Internal Medicine University of Gezira Gezira Sudan; ^4^ Department of Internal Medicine University of Sinnar Sudan

**Keywords:** antinuclear antibodies (ANAs), end stage kidney disease, lupus nephritis, membranoproliferative glomerulonephritis

## Abstract

Membranoproliferative glomerulonephritis (MPGN) has previously been used as an umbrella term to describe a spectrum of hypocomplementemic glomerular diseases, which are rare causes of end stage kidney disease (ESKD). We present a 22‐year‐old man with a well‐established medical history who had been complaining of 4 days of frothy dark urine, bilateral lower limb swelling, and puffiness on his face. For a month before his presentation, he had many bilateral skin lesions on his lower limbs that were leaking pus. Aside from the scars from the prior skin lesions, he had no other significant medical history, and his examination revealed no abnormalities. His tests revealed nephrotic range proteinuria with a normal renal profile, low serum albumin with low C3 and normal C4, and negative antinuclear antibodies (ANAs) by ELISA. After preliminary studies, we concluded that the condition was infection‐related glomerulonephritis. Nevertheless, following renal biopsy, which revealed an MPGN pattern, and immunohistochemistry, which revealed a full house picture, we conducted a second ANA test using the more sensitive/broader spectrum IFA hep2 cell test, which showed a coarse speckled nuclear pattern with a significant titer (1/1000), as well as a negative line blot assay test using 15 distinct antigens. Following the modification of our diagnosis to lupus nephritis, the patient responded fairly well once we started him on an immunosuppressive drug. The patient was released from the hospital in a stable condition.

**Summary**

- Despite recent advances in our understanding of immune‐complex MPGN (IC‐MPGN) and C3 glomerulopathy (C3G), several unmet needs remain in the diagnosis and management of patients with these nephropathies.
- This is partly because of their overlapping clinical presentations, histologic features, and underlying pathophysiologies.
- The presented case emphasizes the importance of rigorous inquiry and careful assessment, since initial indications suggested infection‐related glomerulonephritis, but additional testing, including renal biopsy and immunohistochemistry, revealed the right diagnosis of lupus nephritis.
- This highlights the importance of precise diagnostic approaches in treating complex renal diseases.

## 1 Introduction

Traditional definitions of membranoproliferative glomerulonephritis (MPGN) have outlined a characteristic morphological presentation of glomerular damage, marked by the deposition of electron‐dense immunoglobulins and/or complement elements between endothelial cells and the basement membrane [1]. This process often leads to observable changes under light microscopy, such as thickening of the capillary wall appearing as a double contour (“tram track,” “membranous”) and mesangial cells lodging in a newly developed second layer of the basement membrane (“proliferative”) [2].

Historically, MPGN has been categorized into three types: Types I, II, and III. Type I, the most common form observed in light microscopy, typically displays double contours of the capillary walls and mesangial proliferation. Electron microscopy reveals sub‐endothelial electron‐dense deposits, which may test positive for immunoglobulins or complement factor C3, in both Types I and II [3]. In contrast, MPGN Type II exhibits a unique feature of highly electron‐dense material spread throughout the entire basement membrane, often staining positively for C3 on immunohistology, but usually not or only to a limited extent for immunoglobulins [4]. Presence of sub epithelial immune deposits alongside sub endothelial and mesangial immune deposits are characteristic of MPGN Type III, which can show positivity for immunoglobulins or C3 like in Type 1 MPGN [5].

Asymptomatic hematuria and proteinuria, nephrotic or nephritic syndrome, or even fast progressing glomerulonephritis are some of the clinical manifestations of MPGN. MPGN is progressive and frequently recurs after kidney transplants; if treatment is delayed, it can develop into chronic glomerulonephritis. As a result, uremic toxins are retained, which leads to the development of end‐stage renal disease (ESRD) and chronic kidney disease (CKD), as well as the cardiovascular disorders that are linked to these conditions [1, 6].

The deposition of immune complexes in the kidney can trigger the classical complement pathway, leading to the presence of C1q, C4, and C3 in the glomeruli. Immune‐complex‐mediated MPGN is commonly linked with autoimmune disorders and chronic infections. Notably, infections like hepatitis B and hepatitis C are frequently associated, while systemic lupus erythematosus (SLE) is a prevalent autoimmune disease connected with this condition [7, 8].

A membranoproliferative pattern of injury is often seen in lupus nephritis. MPGN secondary to lupus nephritis typically exhibits more pronounced variations in morphological changes between glomeruli compared to immune‐complex‐mediated MPGN stemming from infections, especially chronic infections [9].

In this communication, we present a case of membranoproliferative glomerulonephritis associated with lupus nephritis in Sudan. We will discuss the clinical and laboratory investigations that were conducted to better understand this unique case and highlight the importance of an accurate diagnosis and appropriate management strategies.

## 2 Case History and Presentation

A 22‐year‐old college student with a well‐established medical history arrived at Omdurman Military Hospital complaining of 4 days of frothy dark urine, bilateral lower limb swelling, and facial puffiness. He began experiencing face and periorbital edema before 4 days, primarily in the morning, and it subsided over the day, for which he took medication for what were thought to be allergies, but with no improvement. He experienced bilateral lower limb edema 2 days later. His brother, a medical officer at the time, conducted urine and blood tests. Following the outcome, he sought medical guidance. When questioned further, he revealed that he had developed a number of itchy skin sores on his legs approximately one month prior, which, when scratched, turned painful and released pus. He did the cleaning himself, and they improved without the use of medications about a week before his presentation. Two days before his presentation, his urine turned frothy and dark, but the quantity and frequency of urination were normal; he did not experience any pain in his loins, suprapubic area, or burning micturition. He denied having a recent upper respiratory tract infection or a history of hematuria. He has no history of renal illness and is not known to have diabetes or hypertension. His father's Polycythaemia rubra vera (PRV) and hypertension are the only positive family history he has.

**FIGURE 1:**
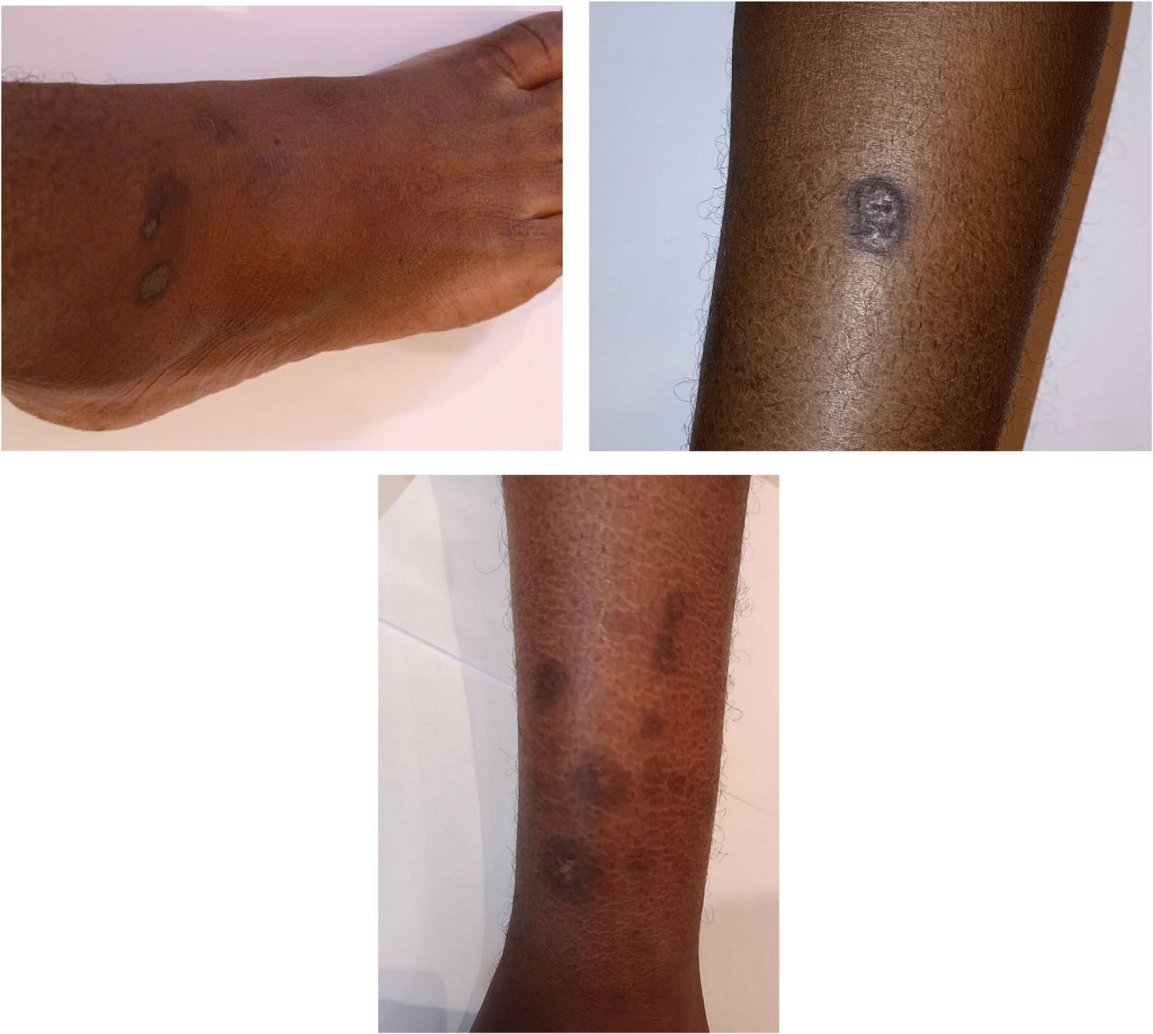
Multiple lower‐limb skin lesions.

Regarding his drug history, he had no allergies; he self‐prescribed hydrocortisone injections and antihistamine tabs for 2 days to manage his facial swelling. Neither a significant social history nor any other noteworthy drug history exists. Vitals on examination included a regular heart rate (HR) of 80 beats per minute, a blood pressure (BP) of 120/70, a respiratory rate (RR) of 14 cycles per minute, a temperature of 36.9, mild peri‐orbital swelling that was neither pale nor jaundiced, a normal jugular vein pressure (JVP), and clear neurological, cardiovascular, chest, and abdomen examinations. He exhibited minor edema in both lower limbs. An examination of his lower limbs revealed many, dispersed scars on his ankles and legs (Figure 1).

## 3 Differential Diagnosis, Investigations and Treatment

The differential diagnoses of this condition included infection‐related glomerulonephritis, C3 glomerulopathy and lupus nephritis. His investigations showed: Urine analysis (on November 10, 2022): Protein: ++++, RBCs: 13–15, Pus: 6–8, Granular cast ++, Urine for albumin/creatinine ratio: 4.012 mg/g; and urine protein creatinine ratio (UPCR): 3.7 mg/mg (reference range: less than 0.2 mg/mg). CBC: HB: 14 g/dL, TWBCs: 7.3 × 10^9^/L, Platelet: 292/mm^3^, renal function tests (RFTs) and electrolytes: blood urea: 37 mg/dL; serum creatinine: 0.9 mg/dL; serum Na: 137 mmol/L; serum K: 4.5 mmol/L; and serum uric acid: 7 mg/dL. Liver function tests (LFTs) and enzymes: total serum protein: 5.2 g/dL; serum albumin: 2.8 g/dL; serum globulin: 2.4 g/dL; total bilirubin: 0.13 mg/dL; ALT: 35 U/L; AST: 34 U/L; ALP: 62 U/L; CRP: negative; ASO titer: negative. Viral screening for HBV, HCV, and HIV: negative, Complement level: C3: 71 (low) reference range (90–180 mg/dL), C4: 11.3 (reference range: 10–40 mg/dL), ANA global by ELISA: negative, a swab from the leg ulcer was taken for culture and sensitivity. As the patient had nephrotic range proteinuria, and hypocomplementemia, renal biopsy was performed which showed 50 glomeruli, which were enlarged, lobulated, and displayed mesangial and endocapillary proliferation. The glomerular basement membrane is thickened with a double contour; there is no sclerosis, thrombosis, necrosis, or crescents seen. The tubules are unremarkable; the interstitium is unremarkable; and the arteries and arterioles are unremarkable.

**FIGURE 2:**
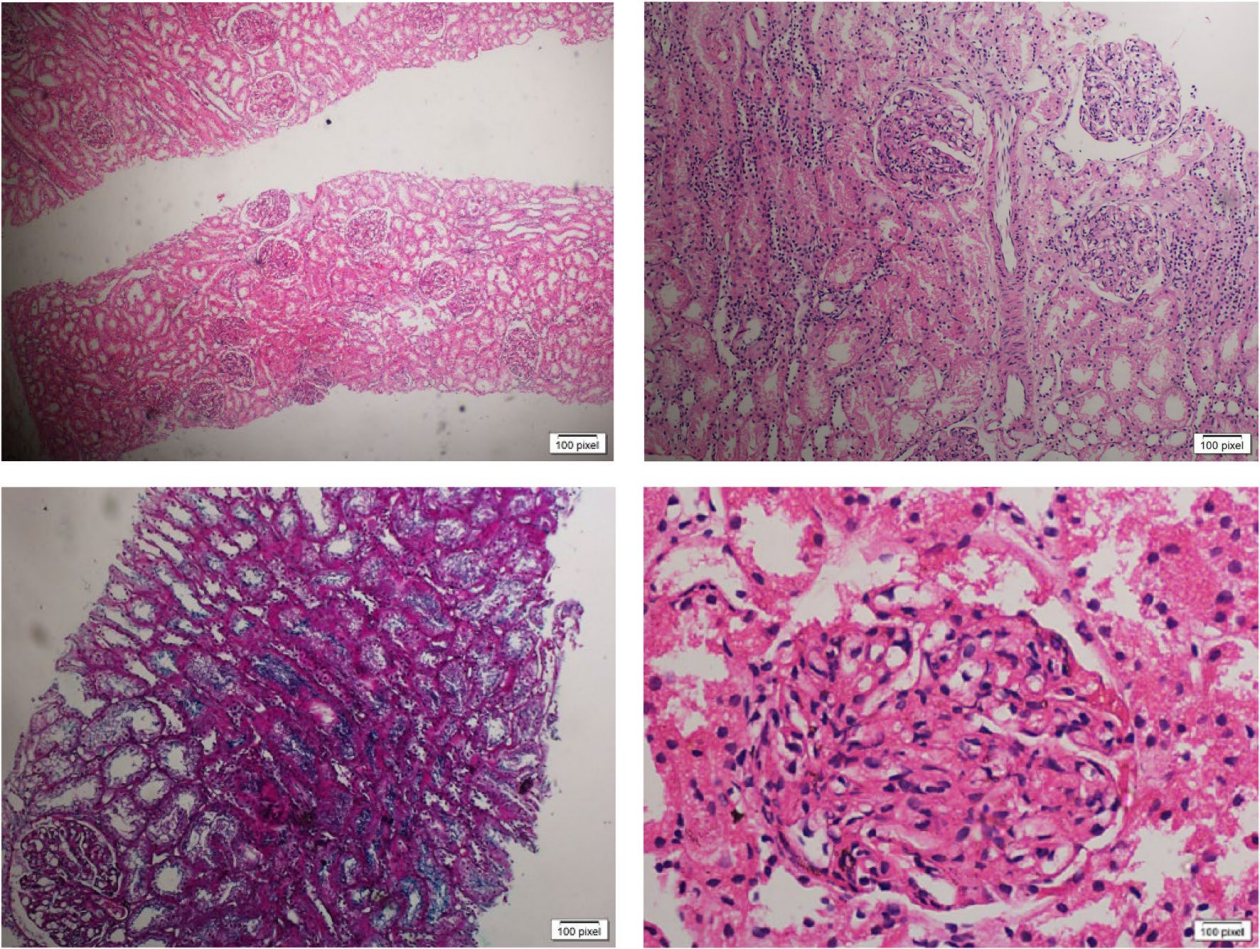
Renal biopsy of the patient showing an MPGN‐like pattern.

The biopsy revealed an MPGN‐like pattern (Figure 2); in view of the low complement, the possibility of immune complex glomerulonephritis was considered. Clinical workup and immunofluorescence for verification: Immunohistochemistry revealed the following: IgG: positive, IgM: positive, IgA: positive, and C3: positive; in view of the low complement and positive immunoglobulins, the possibility of lupus nephritis was considered (for workup and clinical correlation). The swab culture was positive for Methicillin‐resistant *Staphylococcus aureus* (MRSA), which is sensitive to doxycycline and ciprofloxacin. A second ANA test was conducted using the more sensitive/broader spectrum IFA hep2 cell test, which showed a coarse speckled nuclear pattern with a significant titer (1/1000), as well as a negative line blot assay test using 15 distinct antigens (dsDNA, sm, RNP, SS‐A (Ro60 and Ro 52), SS‐B, Scl‐70, PM‐Sc 100, Jo‐1, Centromere B, PCNA, Nucleosomes, Histones, Ribosomal P protein, and AMA‐M2).

Regarding his management, our patient was given oral steroids, culture‐based antibiotics, furosemide, and ACE inhibitors (he started prednisolone tabs at 30 mg per day (0.75 mg/kg/day) 2 weeks after the onset of the disease based on an immunologist consultation done by the patient himself and not by our team).

He performed a urine analysis 1 month later on December 8, 2022, and the results were normal.The urine albumin/creatinine ratio (ACR) was 1.8 mg/g and the serum albumin was 2.8 g/dL. He began reducing his daily dosage of the steroid to 20 mg.

## 4 Outcome and Follow‐Up

The patient was asymptomatic when he was assessed again 6 weeks from the onset of his disease, and both the physical examination and blood pressure were normal. His investigations revealed: Urine analysis: protein +++ and RBCs 18–20, RFTs + electrolytes: blood urea: 24 mg/dL, serum creatinine: 0.6 mg/dL, and serum electrolytes were all within the normal limits. Anti‐dsDNA: negative. Complement level: C3: 110 mg/dL (reference range 90–180); C4: 13 mg/dL (reference range: 10–40). He was taking lisinopril, furosemide, and 20 mg of oral prednisolone daily. Then he was discharged from the hospital in a stable state, and he was referred for a combined clinic (nephrology and rheumatology) for further follow‐up and management.

## 5 Discussion

In this case report, we describe a 22‐year‐old male patient who had lupus nephritis with an MPGN‐like pattern. The patient had multiple bilateral lower limb skin lesions that were draining pus for 4 weeks before presentation, nephrotic range proteinuria with a normal renal profile, low serum albumin with low C3 and normal but borderline low C4, and a coarse speckled nuclear pattern with a significant titer (1/1000) by ANA hep2 IFA. The patient was discharged from the hospital in a stable state after receiving effective treatment with ACE inhibitors, furosemide, culture‐based antibiotics, and oral steroids.

Acute forms of glomerulonephritis (GN) can result either from a primary renal cause or from a secondary disease that causes renal manifestations. For example, *Staphylococcus aureus* infection leads to glomerulonephritis [1]. In this case, the swab culture showed growth of methicillin‐resistant *staphylococcus aureus* (MRSA). However, following the presence of nephrotic range proteinuria in addition to renal biopsy findings, low C3 levels and positive ANA testing using IFA, our diagnosis changed from MRSA‐related glomerulonephritis to lupus nephritis.

There are not any widely recognized categories for glomerulonephritis. Recent developments in our knowledge of the pathophysiological pathways, however, point to the evaluation of genetic analysis, immunological characteristics, and biomarkers [10]. Based on immunofluorescence results from renal biopsies, membranoproliferative glomerulonephritis (MPGN) was reclassified as immune‐complex MPGN (IC‐MPGN) and C3 glomerulopathy (C3G), which shed light on these two different conditions. Based on electron micrographic results, C3G is further divided into C3 glomerulonephritis (C3GN) and dense deposit disease (DDD) [9].

In terms of diagnosis, one should check for an underlying illness in patients with immune complex‐mediated GN (ICGN). Infections like HBV and HCV and chronic bacterial infections (like endocarditis, shunt nephritis, and abscesses) should be taken into consideration first. Secondly, autoimmune disorders like SLE (especially in the chronic phase of lupus nephritis) and, less frequently, Sjögren's syndrome or rheumatoid arthritis should be considered [11].

In terms of therapy, there are three primary approaches to managing a patient with immune‐complex‐mediated MPGN: (a) identifying and treating the underlying disease, (b) evaluating the renal prognosis, and (c) immunosuppressive therapy [10].

The autoimmune condition known as systemic lupus erythematosus (SLE) is primarily diagnosed in young women, with a female‐to‐male ratio of almost 15:1. Up to 75% of patients with SLE may experience kidney involvement (lupus nephritis), one of the most prevalent and severe symptoms of SLE [12]. LN is characterized by various clinical and laboratory features. Understanding these manifestations is crucial for diagnosis and management. Common clinical manifestations include hematuria, edema, and hypertension, consistent with this case. Laboratory manifestations of LN patients include positive anti‐dsDNA, hypocomplementemia (low C3 and C4 levels), and proteinuria [13, 14]. In this case, urine analysis revealed hematuria and nephrotic range proteinuria consistent with the laboratory features of LN. Both lupus nephritis and MPGN can cause hypocomplementemia. In this case, complement activation is met with C3 low and C4 normal but borderline low (3 points), according to the 2019 American College of Rheumatology's (ACR) classification criteria for systemic lupus erythematosus. However, the renal histological traits (class IV LN) that meet the grade criterion of eight points are given the highest weight. Although it is not unique to SLE, proteinuria is a classic criterion and an essential clue. Combining all of these findings with the patient's outstanding reaction to immunosuppressive therapy, we concluded the possibility of LN.

Employing the 2019 ACR criteria, which require 10 points for inclusion, our case fulfilled the entry criterion of being ANA hep2 positive at a titer of 1/1000; In spite of being negative for monospecific antibodies, the case met mainly the clinical domains of the criteria with a full picture of renal involvement. ANA positivity may still suggest SLE because the condition is associated with a broad spectrum of autoantibody reactivity. Like other sm epitopes, the majority of the ribonucleo‐proteins found in the coarse speckled nuclear pattern are recognized to be consistent with SLE. Despite the fact that SLE is more common in women, it is also noteworthy that the patient is male. Given that atypical SLE symptoms might be seen in some cases, it is clear that looking at men is essential. The clinical features, proliferative diffuse GN (lupus nephritis), and positive ANA allowed for the diagnosis of SLE.

## 6 Conclusion

In conclusion, the early presentation of our case had characteristics of GN associated with infection. However, we had to reevaluate our initial diagnosis according to the kidney biopsy results, low C3, and the presence of nephrotic range proteinuria. Therefore, it is important to correlate the clinical picture with the laboratory workup and the histopathological findings in renal disease, as it is not always as it seems.

## Author Contributions

**Ayman Azhary:** conceptualization, data curation, investigation, writing – original draft. **Mohammed Taha:** conceptualization, data curation, formal analysis, investigation, validation. **Nooh Mohamed Hajhamed:** conceptualization, data curation, formal analysis. **Salahaldeen Ismail Mohammed:** writing – original draft, writing – review and editing. **Nouh Saad Mohamed:** investigation, methodology, writing – review and editing. **Waleed Azhary Sir Alkhatim:** investigation, methodology.

## Consent

Written informed consent was obtained from the patient to publish this report in accordance with the journal's patient consent policy.

## Conflicts of Interest

The authors declare no conflicts of interest.

## Data Availability

The data that support the findings of this study are available in the main manuscript of this article.
